# ARISCAT and LAS VEGAS risk scores for predicting postoperative pulmonary complications after cardiac surgery: a cohort study

**DOI:** 10.1097/MS9.0000000000002191

**Published:** 2024-05-20

**Authors:** Khalid M. Siddiqui, Muhammad H. Farooqui, Muhammad S. Yousuf, Muhammad A. Ali

**Affiliations:** Department of Anaesthesiology, Aga Khan University Hospital, Karachi, Pakistan

**Keywords:** ARISCAT risk index score, diagnostic accuracy, LAS VEGAS risk score, open-heart surgery, postoperative pulmonary complications (PPC)

## Abstract

**Background:**

Postoperative pulmonary complications (PPCs) could lead to morbidity, mortality, and prolonged hospital stay. Different risk-scoring systems are used to predict the identification of patients at risk of developing PPCs. The diagnostic accuracies of the Assess Respiratory Risk in Surgical Patients in Catalonia (ARISCAT) and Local Assessment of Ventilatory Management During General Anaesthesia for Surgery (LAS VEGAS) risk scores are compared in prediction of PPCs taking pulmonary complication as the gold standard in cardiac surgery.

**Materials and methods:**

A prospective cohort study with consecutive sampling technique. A total of 181 patients were included. Quantitative data is presented as simple descriptive statistics giving mean and standard deviation, and qualitative variables are presented as frequency and percentages. Sensitivity, specificity, positive and negative predictive values, and diagnostic accuracies are also calculated.

**Results:**

Total 181 post-cardiac surgery patients were analyzed. The median [interquartile range] of age, height, weight, and BMI were 60.0 [52.0–67.0] years, 163.0 [156.0–168.0] cm, 71.0 [65.0–80.0] kg and 27.3 [24.2–30.4] kg/m^2^. 127 (70.2%) were male, and 54 (29.8%) were female. Sensitivity, specificity, positive predictive value, negative predictive value, and diagnostic accuracy of ARISCAT for the prediction of PPCs were (94.9%, 4.65%, 76.1%, 22.9% and 73.4%), whereas LAS VEGAS were (97.1%, 4.65%, 76.5%, 33.3% and 75.1%), respectively.

**Conclusion:**

Both the ARISCAT and LAS VEGAS risk scores are of limited value in cardiac surgery patients for the prediction of postoperative pulmonary complications, based on the predicted scores in this study.

## Introduction

Highlights

- Patients belonged to extensive geographic area that included rural, and urban patients with wide-ranging health and social level.
- Las Vegas and ARISCAT are sensitive risk-scoring tools for the prediction of postoperative pulmonary complication.
- ARISCAT and LAS VEGUS scores for perioperative assessment help in recognizing the patients at a higher chance of developing PPCs.

In modern times, postoperative pulmonary complications (PPCs) play a vital role in patient morbidity, mortality, as well as in the length of stay in hospital. PPCs include bronchospasm, atelectasis, pneumonia, pleural effusion, pulmonary oedema, and pneumothorax. PPC is a very crucial independent component of postoperative 30-day mortality and influences long-term clinical outcomes^1,2^. PPCs are major contributors to the overall risk of surgery and can occur in up to 30% of individuals who undergo it^3,4^. It has been strongly stated that preoperative identification is highly important to appropriately evaluate patients at high risk for developing complications in cardiac surgery. Preoperative risk scores assist in determining postoperative clinical judgement, and a preoperative risk prediction instrument for PPCs could help clinical recognition of patients at increased risk.

This study is a joined use of two prediction tools that is “The Assess Respiratory Risk in Surgical Patients in Catalonia (ARISCAT)” and the “Local Assessment of Ventilatory Management During General Anaesthesia for Surgery (LAS VEGAS)” to predict PPCs. ARISCAT is a well-developed and experienced score with 87.3% sensitivity and specificity of 79.1%^5,6^. Whereas the LAS VEGAS scoring system is a more recent one and it has established moderate and discriminatory performing, for predicting PPCs and it could be beneficial for recognizing individual patients in dire condition^7^.

Both scores are made up of information on the preoperative procedure and preoperative patient variables, and the existence of comorbidities^8,9^ but do not make use of intraoperative events, like those associated with intraoperative ventilation^8^ and systemic circulation^10,11^. Incorporating intraoperative events into prediction models should improve predictability because it has been revealed that they are related to postoperative outcomes^12^.

Our intention is to look at the validated developed prediction score for PPCs, based on preoperative ARISCAT estimated score and LAS VEGAS for intraoperative data. We hypothesized that adding intraoperative data would improve predictability of PPC.

Therefore, the main objective of this study is to compare the diagnostic accuracy of ARISCAT and LAS VEGAS risk-scoring systems in the prediction of PPCs taking pulmonary complication as the gold standard in cardiac surgery.

### Methodology

**Figure 1:**
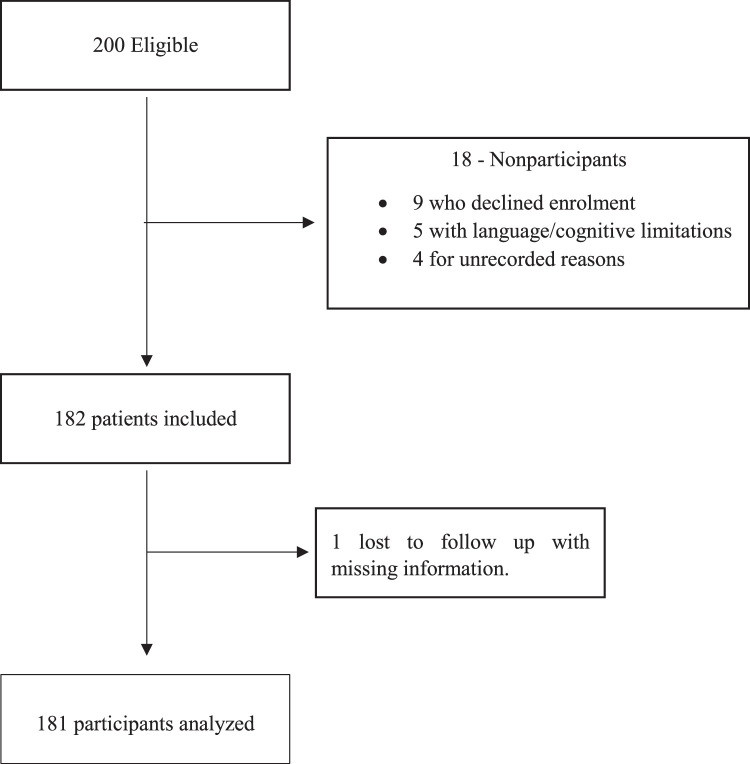
Flow diagram of the study.

This prospective cohort study was conducted from December 2019 to June 2021 after the approval from institutional ethics review committee. A total of 181 patients with either sex, age between 18 and 65 years who underwent elective open-heart surgery were included in the study with a nonprobability consecutive sampling technique. Patients refused to give consent, procedures outside the operating room and emergency surgeries were excluded. The written informed consent was obtained from all patients enroled in the study. The flow diagram of the study mentioned in Figure 1.

Perioperative data was collected in written form in the ward, preoperative area, and intraoperative data was collected in operating rooms. Perioperative characteristic noted on pre-designed proforma, and assessment of risk score was done according to ARISCAT scoring system (Table 1) and intraoperative data has attain giving by LAS VEGAS scoring system (Table 2) by one of the investigators. The cut-off values to ARICAST as greater than or equal to 27 and LAS VEGAS was greater than or equal to 8 were determined for PPC.

**Table 1:** The seven ARISCAT risk predictors

Demographic characteristics	Risk score^a^
Age (year)	
≤50	0
51–80	3
>80	16
Preoperative SpO_2_	
≥96%	0
91–95%	8
≤90%	24
Respiratory infection in the last month	
No	0
Yes	17
Preoperative anaemia (Hb ≤10 g/dl)	
No	0
Yes	11
Surgical incision	
Peripheral	0
Upper abdominal	15
Intrathoracic	24
Duration of surgery (h)	
<2	0
2–3	16
>3	23
Emergency procedure	
No	0
Yes	8

a Independent predictors of risk for PPCs. Three levels of risk were indicated by the following cut-offs: <26 points, low risk; 26–44 points, moderate risk; and ≥45 points, high risk.

ARISCAT, Assess Respiratory Risk in Surgical Patients in Catalonia; Hb, haemoglobin; SpO_2_, arterial oxyhemoglobin saturation by pulse oximetry.

**Table 2:** The thirteen LAS VEGAS Risk predictors

Demographic characteristics	Risk score^a^
Age (year)	
≤46	0
47–67	3
≥68	4
ASA PS	
<3	0
≥3	6
Preoperative anaemia	5
Preoperative SpO_2_	
>96	0
≤96	2
Cancer	5
Obstructive sleep apnoea	9
Surgical characteristics	
Condition of surgery	
Elective	0
Urgency	8
Emergency	9
Duration of surgery (min)	
≤55	0
56–134	4
≥135	11
Intraoperative characteristics	
Use of supraglottic device	−6
Type of anaesthesia	
Totally intravenous	0
Volatile	0
Balanced	5
Desaturation	12
Need of vasoactive drug	5
Mechanical ventilation characteristics	
PEEP (cmH_2_O)	
≤2	0
3–4	3
≥5	4

a Incidence and predicted probability of PPCs according to cut-offs of simplified LAS VEGAS risk score. Low risk,≤7; moderate risk, 8–16; and high risk, ≥17. PPCs.

ASA PS, American Society of Anesthesiologists physical status; LAS VEGAS, Local Assessment of Ventilatory Management During General Anaesthesia for Surgery; PEEP, positive end-expiratory pressure; SpO_2_, arterial oxyhemoglobin saturation by pulse oximetry.

A Pulmonologist having at least five years of experience made the report of postoperative pulmonary complications if any occur like; atelectasis, pneumonia, pleural effusion, pneumothorax, bronchospasm, and pulmonary oedema. These reports were also followed by the primary investigator. PPCs were observed daily from day 0 (surgery day) until discharge from the hospital or until surgery day 5 or, which came first. Each adverse pulmonary event was noted daily but counted once in the composite score. The data regarding socio-demographics was also reported in pre-designed proforma. The work has been reported in line with the STROCSS criteria^13^.

### Statistical analysis

The sample size is estimated by the assumption that the expected sensitivity=87.3% and specificity=79.1% of diagnostic accuracy^5^, while the prevalence for a pleural effusion was 75.6%^14^. It was estimated that 181 patients were required with 5% level of significance and margin of error for sensitivity=12% and specificity=12% (only cardiac surgery).

Statistical analyses were conducted using RStudio software version 4.2.2. Quantitative variables including age, height, weight, and BMI, etc. were summarized as median [interquartile range (IQR)] based on the normality assumption. Qualitative data such as sex, performed procedure, and findings on the ARISCAT and LAS VEGAS scoring systems were presented as frequencies and percentages. To assess the performance of ARISCAT and LAS VEGAS scoring systems in predicting pulmonary complications, a contingency 2 by 2 table was utilized to calculate sensitivity, specificity, positive predictive value (PV), negative predictive value (NPV), and diagnostic accuracy (DA), with pulmonary complications serving as the gold standard. Additionally, diagnostic testing accuracy, including sensitivity, specificity, and predictive values, was also estimated. Multivariable binary logistic regression model was performed to evaluate factors associated with postoperative complication. A level of *P* less than or equal to 0.05 was considered statistically significant.

## Results

Out of 181 patients, the median age was recorded as 60.0 years with an IQR [52.0–67.0]. Median and interquartile range of height, weight, and BMI were 163.0 [156.0–168.0] cm, 71.0 [65.0–80.0] kg, and 27.3 [24.2–30.4] kg/m^2^ are shown in Table 3.

**Table 3:** Demographic data of patients

Variable	Estimate *N*=181
Age (year), median [IQR]	60.0 [52.0–67.0]
Age (year), *n*/total *N* (%)	
15–40	20/181 (11.1)
41–65	161/181 (88.9)
Sex, *n*/total *N* (%)	
Male	127/181 (70.2)
Female	54/181 (29.8)
Height (cm), median [IQR]	163.0 [156.0–168.0]
Weight (kg), median [IQR]	71.0 [65.0–80.0]
BMI (kg/m^2^), median [IQR]	27.3 [24.2–30.4]
Duration of surgery, *n*/total *N* (%)	
≤4 H	10/181 (5.5)
>4 H	171/181 (94.5)
Surgical procedure, *n*/total *N* (%)	
coronary artery bypass graft (CABG)	147/181 (81)
Aortic valve replacement (AVR)	13/181 (7)
Mitral valve replacement (MVR)	19/181 (11)
Ventricular septal defect (VSD)	2/181 (1)

IQR, interquartile range.

PPCs occurred in 137 (75.7%) patients. 72 (39.8%) patients had combined atelectasis and pleural effusion noticed as main complication. Individual atelectasis noticed in 34 (18.8%) patients and pleural effusion in 17(9.4%) patients. Other complications including pulmonary oedema and pneumonia were also noticed single alone and with other complications. A total of 15 (8.2%) patients required incentive spirometry therapy, 6 (3.3%) patients required additional antibiotic cover, and 15 (8.2%) patients required non-invasive ventilation (NPV). No mortality was observed among study patients during hospital stay.

**Figure 2:**
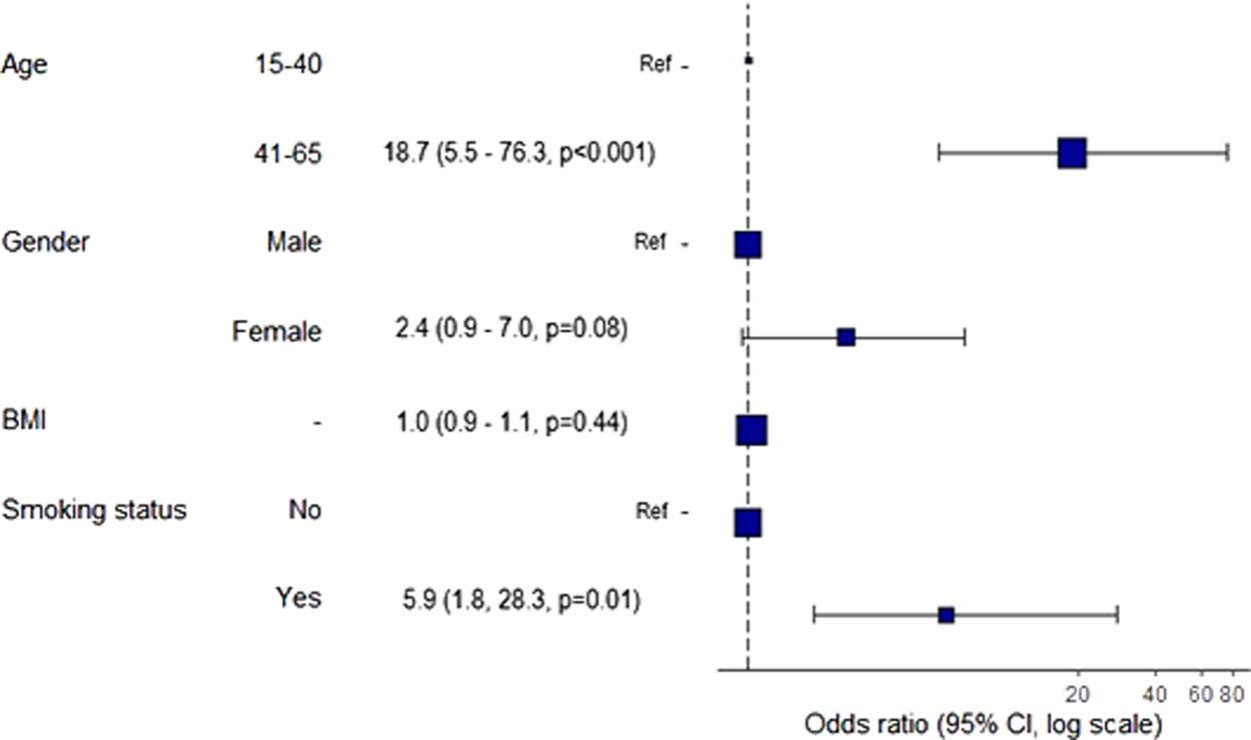
Forest plot of odds ratios of predictors of risk for postoperative pulmonary complications.

In the multivariable binary logistic regression analysis predicting postoperative complications, patients aged 41–65 years had a significantly higher odds ratio (OR) of 18.7 (95% CI: 5.5–76.3, *P*<0.001) compared to the reference group (15–40 years). Female sex showed a non-significant trend towards increased risk (OR: 2.4, 95% CI: 0.9–7.0, *P*=0.08), while smokers were associated with a significantly elevated risk (OR: 5.9, 95% CI: 1.8–28.3, *P*=0.01), compared to non-smokers. Age, BMI, and sex did not show statistically significant associations with postoperative complications (Fig. 2).

The sensitivity, specificity, positive predictive value, negative predictive value, and diagnostic accuracy of ARISCAT score greater than or equal to 27 for the prediction of PPCs development as gold standard was found to be 97.1%, 4.7%, 76.5%, 33.3% and 75.1%, respectively. Table 4

**Table 4:** Diagnostic accuracy of ARISCAT score for the prediction of PPCs

	PPCs		
ARISCAT score ≥27	Yes	No	Total
Yes	134 (TP)	41 (FP)	175
No	4 (FN)	2 (TN)	6
Total	138	43	181
Sensitivity	97.1%		
Specificity	4.7%		
Positive predictive value	76.5%		
Negative predictive value	33.3%		
Diagnostic accuracy	75.1%		

ARISCAT, Assess Respiratory Risk in Surgical Patients in Catalonia; FN, false negative; FP, false positive; PPC, postoperative pulmonary complication; TN, true negative; TP, true positive.

The sensitivity, specificity, positive predictive value, negative predictive value, and diagnostic accuracy of LAS VEGAS score greater than or equal to 8 for the prediction of PPCs development as gold standard was found to be 94.9%, 4.65%, 76.1%, 22.9% and 73.4%, respectively. Table 5

**Table 5:** Diagnostic accuracy of LAS VEGAS Score for the prediction of PPCs

	PPCs		
LAS Vegas Score ≥8	Yes	No	Total
Yes	131 (TP)	41 (FP)	172
No	7 (FN)	2 (TN)	9
Total	138	43	181
Sensitivity	94.9%		
Specificity	4.7%		
Positive predictive value	76.1%		
Negative predictive value	22.9%		
Diagnostic accuracy	73.4%		

FN, false negative; FP, false positive; LAS VEGAS, Local Assessment of Ventilatory Management During General Anaesthesia for Surgery; PPC, postoperative pulmonary complication; TN, true negative; TP, true positive.

## Discussion

This is the first prospective observational study, as per the literature review in which both ARISCAT and LAS VEGAS scoring systems are evaluated together with their diagnostic accuracies to predict PPCs after cardiac surgery. In this study both LAS VEGAS score greater than or equal to 8 and ARISCAT score greater than or equal to 27 are practical and reasonable risk-scoring tools for the prediction of PPCs undergoing cardiac surgery with acceptable accuracies. Mostly, pulmonary complications after cardiac surgery include pleural effusions, atelectasis, pulmonary/cardiogenic pulmonary oedema, pneumonia, mediastinitis and pneumothorax, with different outbreaks in patients have been reported. Atelectasis is known to be a common complication found after cardiac surgery, which causes impaired gas exchange and hypoxaemia. Atelectasis is reported in literature about 30–72% in post-cardiac surgery and considered as the main contributor to the postoperative respiratory dysfunction^15^. In this study atelectasis is found in the majority of patients but does not cause any major respiratory impairment and only 15 patients required non-invasive ventilation (NIV) for 12–36 h duration, and no patient required reintubation and mechanical ventilation.

By virtue of the nature of cardiac surgery, thoracic cavity intervention is a significant contributor to the increase of PPCs. Furthermore, these patients’ existing clinical conditions can often deteriorate, leading to numerous unstable laboratory parameters. Cardiac surgery itself is considered as with higher ARISCAT parameters, and usually preoperative ARISCAT scores are noted intermediate to high grade in cardiac surgeries. Specially, we have particularly evaluated the effectiveness of the LAS VEGAS scoring system, which is found to be an effective parameter to determine the diagnostic accuracies for PPC in cardiac surgeries besides ARISCAT risk score index when using together. Previous studies have been shown that geriatric age group is an important factor in the development of PPCs^4^. Additionally, it has been noted that the male gender age ≥65 years and duration of surgery greater than or equal to 2.5 h are potential risk factor for the occurrence of PPCs. Notably The LAS VEGAS risk score displayed many of the same criteria as the ARISCAT risk score, but it also had some notable differences, such as intraoperative complications and obstructive sleep apnoea, which may have contributed to its better presentation. However, it is important to note that The LAS VEGAS risk score is also considered intraoperative variables, whereas the ARISCAT risk score has a preoperative value. Certainly, the ability of a score to predict an event is higher when you are closer to the event^16,17^. In our study, the results are consistent with the literature; male gender, advancing age, and duration of surgery are the predictable factors in the development of PPCs.

Conventionally the American Society of Anesthesiologists (ASA) physical status classification system is consistently used to individually predict preoperative health condition. The ASA physical status is still insufficient for calculating perioperative risk, though. Consequently, it prompted questions regarding the scale’s reliability^18^. This reveals that more broad preoperative risk assessments are needed in surgeries, especially cardiac surgery where perioperative complications are more widespread. The ARISCAT and LAS VEGAS risk index can provide complete results by assessing many factors. However, our study is unique in this regard, since studies on ARISCAT risk assessment generally cover diverse surgical groups in the past. The association between PPCs and ASA physical status has previously been studied by Kupeli *et al* ^19^, who found that ASA physical status is a poor method in predicting the PPCs. They also assessed the association between the ARISCAT risk score and PPCs in their study, concluding that the ARISCAT risk index provided a more accurate prediction of PPCs.

In this study the LAS VEGAS risk score performed relatively better. But it is important to understand the possible reasons for the slightly reduced predictive performance of the ARISCAT score in the present study compared with the previous assessments. In high-risk patients, ARISCAT score seems to have a better predictive value, but the addition of intraoperative variables of LAS VEGAS score could identified PPC in the patients marked with lower risk preoperatively.

Finally, when using the intraoperative data, the level of positive end-expiratory pressure (PEEP) level is a very important factor during intraoperative ventilation. PEEP levels of 5 cmH2O or less are typically used, which is consistent with reports from previous studies^20–23^. As LAS VEGAS score parameter, no association between PEEP levels and the occurrence of PPCs is found in this study, which is consistent with previous trial^24^. The LAS VEGAS risk score must consistently be utilized in its whole shape; the effects of only one or two variables should never be considered.

This study was done in a tertiary care centre, and the collection of data by the ARISCAT and LAS VEGAS risk-scoring systems from patients undergoing specific anaesthetic and surgical procedures are the strengths of this study. The study also addresses an important clinical issue by evaluating risk-scoring systems for predicting PPCs after cardiac surgery, which can significantly impact patient outcomes. The prospective design using consecutive sampling enhances the validity of the study findings. Additionally, using both pre- and intraoperative data has enhanced the predictability of PPCs.

### Limitations

The study has certain limitations including a single-centre study with quite a small sample size. Additionally, as other independent risk factors that may enhance the risk of PPCs, such as obesity, smoking, and anaemia, were not examined in detail, there is a chance that unmeasured variables will have a confounding influence. Herewith, we did not assess the risk factors for PPCs independently, and we have not followed long-term survival as we reviewed only in-hospital mortality. The study also lacks comparison with other established risk-scoring systems due to external validation needed to confirm the accuracy of the LAS VEGAS score for predicting PPCs after cardiac surgery, which could provide a more comprehensive evaluation. Therefore, more future studies may be required to cover this issue.

## Conclusion

With the stated predictive values, both ARISCAT and LAS VEGAS risk scores are of very limited use in cardiac surgery patients for the prediction of postoperative pulmonary complications in patients undergoing open-heart surgery. Appreciation of outlined risk factors and routine combined use of ARISCAT and LAS VEGAS scores for perioperative assessment may help recognize the patients at a higher chance of developing PPCs. Overall, this study contributes to the literature by evaluating the diagnostic accuracies of both risk scores in predicting PPCs after cardiac surgery. Despite its limitations, the study underscores the potential utility of these risk-scoring systems as screening tools for identifying patients at high risk of PPCs. Further research is warranted to validate these findings and explore additional risk factors for PPCs in this patient population.

## Ethical approval

This Cohort study was conducted from December 2019 to June 2021 after the approval from institutional ethics review committee. (ERC 2020-1743-8434)

## Consent

Written informed consent was obtained from the patient for publication and any accompanying images. A copy of the written consent is available for review by the Editor-in-Chief of this journal on request.

## Source of funding

The authors received no financial support for this study.

## Author contribution

K.M.S. conceived the idea or hypothesized for research and manuscript, supervised the course of the project and constructed the whole body of the manuscript. M.H.F. execution of the study, including informed consent, patient follow-up, data management and reporting. M.S.Y. interpretation and presentation of the results. M.A.A. did literature search and editing of the manuscript. All authors reviewed and provided substantial input to the manuscript.

## Conflicts of interest disclosure

The authors declare no conflict of interest.

## Research registration unique identifying number (UIN)

The study is registered in *Clinicaltrials.gov* as an observational study. UID NCT06173583.

## Guarantor

On behalf of all the contributors, Dr Khalid Maudood Siddiqui will act as guarantor and will correspond with the journal from this point onward.

## Data availability statement

Data will be made available upon a reasonable request to the corresponding author.

## Provenance and peer review

Not commissioned, externally peer-reviewed
